# Management of Postpartum Hemorrhage in Low- and Middle-Income Countries: Emergency Need for Updated Approach Due to Specific Circumstances, Resources, and Availabilities

**DOI:** 10.3390/jcm13237387

**Published:** 2024-12-04

**Authors:** Gulzhanat Aimagambetova, Gauri Bapayeva, Gulnara Sakhipova, Milan Terzic

**Affiliations:** 1 Department of Surgery, School of Medicine, Nazarbayev University, Astana 010000, Kazakhstan; milan.terzic@nu.edu.kz; 2 Clinical Academic Department of Women’s Health, CF “University Medical Center”, Astana 010000, Kazakhstan; gauri.bapayeva@gmail.com; 3 Department General Practitioners, West Kazakhstan Medical University, Aktobe 030000, Kazakhstan; gulnara.7110@mail.ru

**Keywords:** postpartum bleeding, postpartum hemorrhage, maternal mortality, postpartum hemorrhage management, LMICs

## Abstract

Postpartum hemorrhage (PPH) represents a critical emergency condition and the principal cause of maternal morbidity and mortality worldwide. It encompasses excessive bleeding following childbirth, which can arise from various causes. Prompt recognition and management are essential to mitigate severe outcomes and ensure maternal safety. The incidence of PPH in low- and middle-income countries (LMICs) is higher than in developed countries. Healthcare systems in developing countries face multiple challenges that may impact PPH management at policy, facility, and community levels. The mentioned barriers could be addressed by providing an empowering environment via the implementation of supportive policies, access to PPH care, planning supplies, allying strategies, providing training, and utilization of guidelines and algorithms for PPH management. Evidence-based international guidelines should serve as an integral part of appropriate management. On the other hand, LMICs have limited opportunities to implement the proposed international algorithms and guidelines. Therefore, some amendments based on the resource/expertise availability should be considered at the specific clinical site. This review summarizes and updates the accumulated knowledge on postpartum hemorrhage, focusing on challenging management options in developing countries. In many LMICs, maternal morbidity and mortality linked to PPH were improved after the implementation of standardized protocols and timely and purposeful interventions. International support in healthcare professionals’ training, enhancing resources, and the provision of an adapted evidence-based approach could assist in improving the management of PPH in LMICs. Refining our understanding of specific local circumstances, international support in specialists’ training, and the provision of evidence-based approaches may assist in improving the management of PPH in LMICs and contribute to safer childbirth.

## 1. Introduction

Postpartum hemorrhage (PPH) is a dangerous and potentially life-threatening obstetric emergency [1], which is largely underestimated [2]. PPH can lead to severe anemia and disseminated intravascular coagulopathy, both requiring blood transfusion. It may also result in hysterectomy, multisystem organ failure, and death [3,4]. Unfortunately, although frequently preventable and manageable, PPH remains the primary cause of maternal mortality globally [1,2,5]. Inconsistencies in the definition of PPH resulted in an underestimation of the prevalence worldwide [2].

Multiple causes and risk factors are involved in the etiology and pathogenesis of the condition. All pregnant women after 20 weeks of gestation should be considered at risk for postpartum bleeding. While women without risk factors could develop a PPH [6], women who develop PPH usually have some predisposing conditions that are considered risk factors. Thus, PPH rates and the related consequences can be reduced by identifying patients at risk and increasing awareness of the possible complications [3,7].

As acknowledged by the World Health Organization (WHO), there is a growing valuation of quality in healthcare for all patients. Due to specific demands, the quality of health services for pregnant patients should be timely, safe, effective, patient-centered, integrated, and efficient. However, inequalities in healthcare exist between countries in the world due to differences in income, political situation, and cultural specifics. Inefficient healthcare in low-income countries could be a result of ineffective medical education and healthcare organizations, leading to insufficient access to up-to-date medical knowledge and resulting in inadequate training of healthcare professionals. In some cases, there are problems with staffing and a deficiency in qualified midwives and nurses, which leads to low quality of care.

Although the WHO has developed a guideline on PPH prevention and contemporary management, mortality associated with postpartum bleeding has decreased only in high-income countries, but the implementation of these recommendations did not result in a significant decrease in PPH-related morbidity and mortality in developing countries [8]. Additionally, there is an existing roadmap for reducing PPH in 2023–2030, but there has been little progress on this matter, especially in low- and middle-income countries (LMICs) [8].

Considering that PPH remains an up-to-date issue in obstetrics, this review aims to summarize and update the accumulated knowledge on postpartum hemorrhage, its pathogenesis, complications, and management with a focus on challenging management options in developing countries. A better understanding of pathogenesis and treatment might open new opportunities for preventing and better managing PPH and the related adverse outcomes.

## 2. Material and Methods

**Figure 1:**
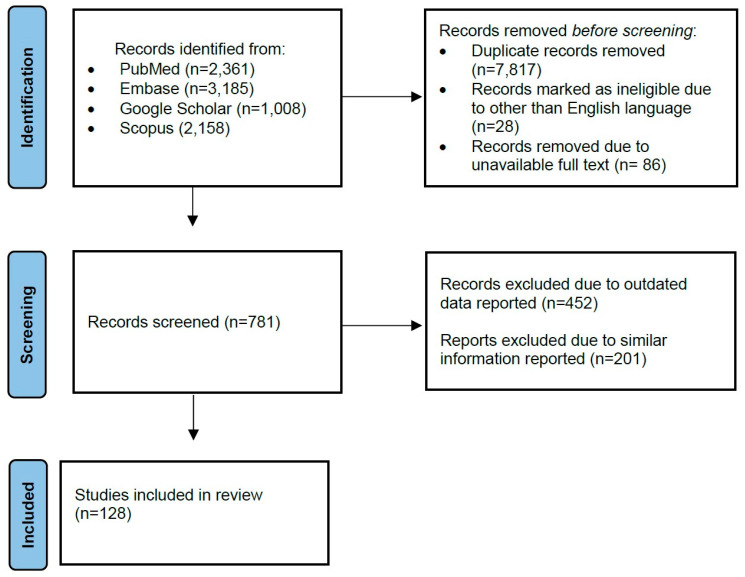
Data collection flowchart.

A literature review was performed by searching the available data on PPH and management, focusing on PPH in the developing world. The data search was conducted in PubMed, Scopus, Web of Science, Embase, and Google Scholar databases for the past 20 years up to the current time (June 2024). The following keywords, combinations of keywords, and MeSH IDs (if available) were searched: “postpartum hemorrhage”, “postpartum bleeding”, “immediate postpartum hemorrhage”, and “delayed postpartum hemorrhage” (MeSH Unique ID: D006473); “management”; “guideline” and “practice guideline” (MeSH Unique ID: D016431); “developing country”, “low-income country”, “middle-income country”, and “developing nations” (MeSH Unique ID: D003906); “obstetrical complication” (MeSH Unique ID: D007744), “prognosis” (MeSH Unique ID: D011379). Peer-reviewed articles relevant to the subject of the study have been read and included in the review (Figure 1). Due to the heterogeneity of the studies and outcomes found, a non-systematic (narrative) review type was conducted. This narrative review could be useful for researchers, medical educators, and students. While a systematic review of PPH should have been focused on a narrow question in a specific context, this narrative review includes a wide variety of studies and provides an overall summary with interpretation and critique [9].

## 3. Results and Discussion

### 3.1. Definitions and Classifications of Postpartum Hemorrhage

#### 3.1.1. Definitions

Various definitions are used to define PPH [2,10]. Characterizations are based on the volume of blood loss, hematocrit levels, hemodynamic changes, and other symptoms [2,11]. Moreover, definitions of PPH vary between guidelines, medical specialties, and countries [2,10].

The simplest and widely used definition that was utilized as a background for more comprehensive definitions is the one stated by the WHO in 1990, which is “any blood loss from the genital tract during delivery above 500 mL” (Table 1) [2,12].

According to the American College of Obstetricians and Gynecologists (ACOG), PPH is defined as “cumulative blood loss greater than or equal to 1000 mL or blood loss accompanied by signs or symptoms of hypovolemia within 24 h after the birth process (includes intrapartum loss) regardless of route of delivery” (vaginal or cesarean section) [10,11,13,14,15,16,17]. The other widely used “traditional” definition of PPH is based on an estimated blood loss that exceeds 500 mL after a vaginal delivery or a loss of greater than 1000 mL after cesarean delivery [18,19,20]. The Royal College of Obstetricians and Gynaecologists (RCOG) suggested a different definition of PPH “as minor (<1000 mL) or major (≥1000 mL)” [10,21].

**Table 1:** Definitions of postpartum hemorrhage.

Guideline	Year	Definition	References
“Traditional” definition	-	- blood loss from the genital tract during delivery above 500 mL after vaginal delivery; - blood loss from the genital tract during delivery above 1000 mL after cesarean delivery	[18,19,20]
WHO	2012	- blood loss of 500 mL or more within 24 h after birth	[11]
ACOG	2017	- cumulative blood loss greater than or equal to 1000 mL regardless of route of delivery	[15]
RCOG	2017	- minor blood loss—<1000 mL; - major blood loss—≥1000 mL	[21]

However, all mentioned definitions are based on the quantitative measurement of blood loss and often appear to be subjective due to many factors [2]. As the amount of blood loss is usually assessed visually, this method is very subjective, with underestimation occurring in up to 50% of cases [22]. The benchmark approach to measuring blood loss is a gravimetric method [17,23]. However, the method is difficult to apply in practice as it is time-consuming and not convenient in cases of PPH, which requires urgent actions. Moreover, the required devices/tools might not be available in low-resource settings [17]. Therefore, the existing definitions are based on alternative methods (in many cases subjective) of measuring blood loss [2]. 

In an alternative description, PPH is defined as a “blood loss sufficient to cause hypovolemia, a 10% decrease in hematocrit or requiring transfusion of blood products regardless of the route of delivery” [24].

Although numerous definitions for PPH have been proposed, none of them appear to be satisfactory and objective. A more unified and standardized definition of PPH is required for obstetrical practice, appropriate for both high- and low-resource settings/countries [2,22]. Thus, the quantification of blood loss and definitions of PPH based on it tends to underestimate PPH [2]. The definition of PPH has a great practical implication as the bleeding management is based on the volume of blood loss.

#### 3.1.2. Classifications

A variety of classifications were developed to describe PPH, including time, clinical signs, laboratory findings, etc. These classifications serve as a guide to actions in PPH, which is considered a true obstetric emergency.

Based on the timing of bleeding, PPH is classified as immediate/primary/early (within the first 24 h of delivery) and delayed/secondary/late (more than 24 h after delivery but less than 12 weeks) [2,5,11,16,20,25]. Depending on PPH’s relation to the labor/delivery stages, bleeding could be described as the second or third stage, depending on whether it occurs before or after delivery of the fetus and placenta, respectively [20].

The suggested RCOG definition of PPH can be used for the classification of the condition as minor and major bleeding, <1000 mL and ≥1000 mL, respectively (Table 2) [5,10,21,26].

Other guidelines also classify PPH into minor (500–1000 mL) and major (≥1000 mL) [10,18]. According to these sources, major PPH could be further subdivided into moderate (1000–2000 mL), severe (>2000 mL) bleeding, and life-threatening blood loss [10,21,22].

### 3.2. Epidemiology of Postpartum Hemorrhage

#### 3.2.1. Prevalence of Postpartum Hemorrhage Worldwide and in Developing Countries

Postpartum bleedings are estimated to complicate 3–13% of births worldwide, with substantial variation across countries [7,14,20,27,28]. The prevalence of PPH may differ due to variations in the assessment method and ranges from 7% (if assessed with subjective techniques) to 12% (if measured by an objective appraisal of blood loss) [2,14,20,21,27]. Severe PPH is estimated at 1–3%, with wide variations across countries depending on their income and the presence of healthcare systems [5]. 

Studies have noted increasing rates of PPH in developed countries during the past two decades [20,29,30]. These reports are coming from Norway [29], Denmark [31], Sweden [32], Canada [33], and the United States [34]. The most recent study from the United States reported an increased rate of PPH in the period of 2000–2019 from 2.7% to 4.3% (AAPC 2.6%, 94% CI 1.7–3.5%) [34]. That might be related to the amplified rates of first and repeated cesarean delivery, increased utilization of artificial reproductive technology (ART), and overall urbanization with adverse health effects, etc. However, the exact causes and factors underlying the increase remain unclear [20,22].

The magnitude of PPH varies between developing countries, largely depending on income. Even within the same country, variation might occur between urban and rural areas, highlighting inequality in healthcare access and quality of care. In particular, in Ethiopia, the incidence of PPH ranges from 1.4% to 16.6% [24]. The pooled magnitude of PPH in Ethiopia was reported as 8–11% [24,35]. In Sub-Saharan Africa, where the maternal mortality rates are highest compared to other world regions, PPH prevalence is reported to complicate from 12% [27,36] to 25.7% of births [28].

Moreover, Latin America and the Caribbean region (3.3%), North America (4.3%), and Africa (5.1%) present the uppermost prevalence rates of severe PPH (≥1000 mL). In Latin America, PPH is the most frequent cause of maternal loss, and the rate is up to 23% [37]. In Asia, the rate of severe PPH is reported to be 1.56% [38].

As seen from the presented data, there is a huge difference in the prevalence of PPH across developing countries. This could be related to a variety of factors, including racial and ethnic differences leading to genetic diversity, social and cultural specifics of countries, availability of resources, differences in healthcare systems and their functioning, etc. In the end, due to multiple factors and obstacles related to limited resources, knowledge, and inappropriate management, pregnant women in low-income countries are at high risk for mortality from PPH [14].

#### 3.2.2. Contribution of Postpartum Hemorrhage to the Incidence of Maternal Mortality

An estimated 14 million cases of PPH occur annually worldwide, and one woman dies every four minutes due to PPH [7]. Thus, PPH is a leading cause of maternal mortality and accounts for up to 25–52% of maternal deaths worldwide, depending on the country’s income, with higher rates in low-resource settings [7,14,24,39,40,41]. In high-income countries, PPH was the main cause of 8% to 13–19% of maternal mortality cases, while in developing countries, it increases up to 25–52% [2,5,7,14,42,43,44,45]. When developed countries are compared with LMICs, wide differences in the incidence of PPH-related deaths become evident [17]. For example, PPH accounts for 11% of “pregnancy-related deaths” in the United States [14] and 19% in Japan [45], while in Sub-Saharan Africa, PPH accounts for 50–52% of maternal mortality cases [27,36].

The real incidence of maternal mortality due to PPH is certainly much higher as many cases remain unreported, especially in developing countries, due to multiple reasons (“blaming” culture, low awareness, etc.). The absolute risk of death is “lower in high-income countries with an estimated rate of 1:100,000 deliveries” compared to “an estimated rate of 1:1000 in low-income countries” [2,7].

Data from some developing countries are controversial. Namely, studies from Ethiopia reported that 46.5% to 54% of maternal mortality cases were caused by PPH [24,46,47]. Data from West Africa reported 32% of maternal mortality cases being associated with PPH [40,48]. On the other hand, the Bureau of National Statistics of the Republic of Kazakhstan and the Ministry of Health reported that PPH accounts for only 5.5–15% out of all cases of maternal mortality [49], which is lower than in high-income countries and any other developing countries, which is inconsistent with the available worldwide statistics, and supports the notion that in many countries PPH cases are underestimated and the reporting systems must be standardized.

### 3.3. Etiology and Risk Factors of Postpartum Hemorrhage

**Figure 2:**
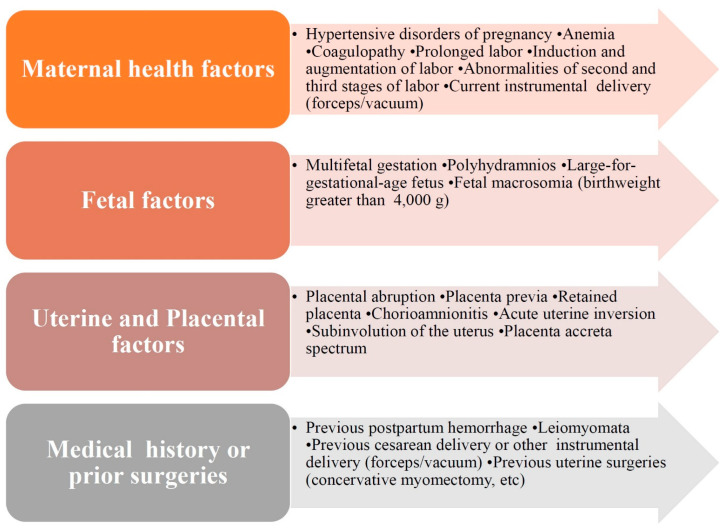
Etiology and risk factors of postpartum hemorrhage.

At least 90% of maternal mortality cases from PPH are avoidable “because they often follow a delay in diagnosis, management, or insufficient treatment” [50]. Multiple causes and risk factors could be responsible for PPH onset and progression and contribute to its pathogenesis (Figure 2). Moreover, PPH can occur in women without risk factors for hemorrhage [6]. Among the main etiology of PPH, four main reasons could be defined: uterine atony (“tonus”), retained placental tissues in the uterine cavity (“tissue”), tissue lacerations (“trauma”), and coagulation disorders (“thrombin”) [6,24,51]. These factors constitute the “4 T” mnemonic/rule to be considered as a PPH cause [3,6].

One of the major etiologies is uterine atony, which is responsible for more than 50–80% of PPH cases [3,6,7,22,24]. PPH associated with uterine atony is often linked to chorioamnionitis in labor, preeclampsia with magnesium sulfate administration, induction or augmentation of labor, uterine leiomyoma, and uterine overdistension (multifetal pregnancy, fetal macrosomia, and polyhydramnios) [3]. Investigating optimal time of amniotomy during labor induction, the most recently published RCT found that delayed amniotomy (artificial rupture of membranes performed ≥ 4 h after Foley balloon removal or expulsion) is linked with PPH, but the underlying potential causal mechanisms should be precisely explored [52].

Uterine atony as the main etiology is followed by obstetrical lacerations responsible for around 20% of PPH, then retained placental tissue—10%, and coagulation disorders—<1% [3,24].

The mode of delivery plays a major role as cesarean delivery is linked to a higher risk of PPH than natural vaginal delivery [7,22]. Obstetrical lacerations (“trauma”) can be caused by precipitous delivery, operative vaginal delivery, or episiotomy [3,7,22]. PPH associated with “tissue” (retained placental and membranes in the uterus) is usually caused by abnormal placentation, including placenta accreta, placenta increta, and placenta percreta [3,7,53,54]. Maternal coagulopathy resulting in PPH could be a complication of preeclampsia/eclampsia, placental abruption, placenta previa, intrauterine fetal death, or any type of preexisting coagulopathy [3]. Maternal age and body mass index (BMI) extremes are also among the risk factors for PPH [3].

In addition to the general etiologies and risk factors, pregnant women in LMICs could suffer from inappropriate and inefficient interpretation of signs related to “4Ps” and further development of PPH. This could result from a low level of training and might contribute to the increasing incidence of PPH.

However, still, a substantial proportion of PPH occurs in the absence of recognized risk factors; thus, any pregnant woman should be considered for risk of PPH in labor [7]. Various tools have been developed to predict PPH [55,56,57,58]. However, studies report a diverse prediction ability of those tools ranging from poor to moderate [55,56]. Thus, more and “better PPH risk stratification tools are required with the inclusion of additional important variables” [56].

Obstetricians working in LMICs, in resource-restricted settings, in many cases are unable to follow international guidelines’ recommendations on PPH due to limited resources and are forced to choose low-cost therapies and procedures [59] immediately.

### 3.4. Assessment Tools for Postpartum Hemorrhage

#### 3.4.1. Blood Loss Assessment

Various methods are used to assess blood loss volume in PPH: visual assessment, quantification methods, and calculation using different equations. Since most PPH definitions are based on the blood loss volume and visual estimation always leads to underestimation of blood loss, objective assessment of bleeding is one of the major tasks in cases of PPH [60]. To make the blood loss evaluation more reasonable, it is strongly recommended to count utilized medical materials (surgical wipes and towels) [22,61,62]. Moreover, using a calibrated collector bag(s) is suggested for a more accurate blood loss volume estimation [60,63]. However, the cost of calibrated collector bag(s) could be an obstacle for some low-income, underdeveloped countries, especially considering that many of them have high birth rates with increasing demand for the maternity healthcare sector.

A gravimetric blood loss measurement based on weighing bags with blood and blood clots lost after delivery could be applied [22]. Quantitative methods of measuring obstetric blood loss are suggested by ACOG as “more accurate than visual estimation in determining obstetric blood loss” [62]. According to ACOG, implementation of a quantitative evaluation of blood loss includes “(1) use of direct measurement of obstetric blood loss (quantitative blood loss) and (2) protocols for collecting and reporting a cumulative record of blood loss postdelivery” [62].

Moreover, the WHO guideline on PPH recommends a routine objective measurement of postpartum blood loss, which further will enable improving the detection and prompt treatment of PPH [60]. Calibrated drapes are suggested as a method to objectively quantify blood loss [60].

Pictorial guidelines are developed to help with the measurement of blood volume loss in PPH. These are especially useful in LMICs when other methods are not available. According to a pictorial guide that assists with “visual estimation of blood loss”, the amount of blood estimations for a smeared sanitary towel is 30 mL, for a soaked sanitary towel is 100 mL, a small soaked swab is 60 mL, a large soaked swab is 350 mL, and a full kidney dish is 500 mL [64,65]. Unfortunately, there is a difference in blood loss measurement and estimation between healthcare professionals (anesthesiologists, obstetricians, midwives, etc.), with obstetricians prone to underestimate blood loss [64]. Blood loss measurement is especially critical in the first two hours after delivery, and all women have to be “regularly monitored for early warning signs of excessive blood loss”, such as tachycardia and/or hypotension [60].

Apart from the weighting methods, a modified Brecher’s formula can be utilized. It relies on hemoglobin measurement after delivery, which makes the assessment more evidence-based and accurate [22,66,67]. In general, calculated blood loss using different formulas was found to be “significantly but moderately” correlated with quantitative measurement after delivery [68].

#### 3.4.2. Postpartum Hemorrhage Risk Assessment

Several risk assessment tools have been developed to forecast and evaluate the risk for PPH, and many of them have been proven to be useful in PPH prediction for women at high risk for PPH [69]. To evaluate the risks of bleeding in childbearing women, researchers from Rwanda developed a content-validated risk assessment tool for PPH prediction and prevention (RATP) [70]. It could serve as an instrument to assess PPH risks in LMICs. Furthermore, the researchers continue their studies to develop predictive models to identify high-risk women for PPH and direct the implementation of targeted interventions [71].

Various predictive models (logistic regression, logistic regression using an elastic net, Random Forests, Extremely Randomized Trees, etc.) are being investigated in trials for predicting PPH [71].

The other options in use as assessment tools for PPH include the Association of Women’s Health, Obstetric and Neonatal Nurses (AWHONN), California Maternal Quality Care Collaborative (CMQCC), and New York Safety Bundle for Obstetric Hemorrhage (NYSBOH) [55,69,72,73]. The CMQCC found that “underestimating blood loss and relying on vital signs led to health care providers underestimating the severity of PPH”, which subsequently resulted in treatment delays [26,73,74]. Some of these tools were found to be useful in predicting PPH among patients at low risk [55]. With the development of artificial intelligence and machine learning, more predictive models could be developed to predict and prevent PPH. The discussed risk assessment tools are suggested for use in LMICs to predict and reduce risks of PPH.

The shock index is another assessment tool that may help in the identification of women with hypovolemic shock [75]. The shock index comprises the ratio of heart rate and systolic blood pressure, which shows a good correlation with blood loss in pregnant and puerperal patients. The shock index calculation is a method to assess hypovolemic shock and PPH severity and could be instrumental to the therapeutic choice [75,76]. Thus, the shock index and grade of shock have been taken into consideration by the obstetrics specialists in the decision-making process regarding the management choice. Patients with mild shock signs could be treated conservatively, whereas, for women with moderate and severe shock, hysterectomy should be considered [76]. Studies confirmed the diagnostic values of the shock index and heart rate [75,76,77]. A statistical significance of these parameters in predicting PPH with “high specificity but low sensitivity” was reported [77].

However, some researchers reported controversial findings. One of the recent systematic reviews on the predictive accuracy of the shock index for severe postpartum hemorrhage in high-income countries revealed that “the predictive performance of the shock index for severe PPH is inconsistent and that its use in clinical settings is uncertain” [78].

Nevertheless, the calculation of the shock index does not require additional resources; only knowledge and training of obstetric specialists and midwives are required. Thus, the method is applicable for PPH evaluation in LMICs.

### 3.5. Prevention and Management of Postpartum Hemorrhage

Considering the risk of morbidity and mortality associated with PPH, important management guidelines have been developed by different obstetrics and gynecology societies and healthcare regulatory bodies [21,60,79,80,81]. All guidelines acknowledge risk factors and are consistent with preventive measures and recommendations for PPH management. Moreover, some of these guidelines consider special recommendations for low-resource settings [80].

For PPH caused by uterine atony, the guidelines recommend the administration of uterotonics for prevention and treatment of PPH and timely surgical management [21,79,80,81]. Some minor inconsistencies are reported regarding the optimal regimens for uterotonics used and dosages depending on the particular uterotonics availability and country-related income and guidelines [18,82,83,84]. If uterotonic therapy fails, the use of a variety of surgical interventions is unanimously recommended.

There is a consensus regarding the management of PPH due to placental factors, perineal lacerations, and coagulation disorders [1,18,82]. Some discrepancies are present regarding the effect of uterine massage as a prophylactic measure for PPH. According to the RCOG and FIGO guidelines, which refer to the Cochrane review [85], uterine massage has no benefit in the prophylaxis of PPH [21] in women “who have received prophylactic oxytocin” [80]. This is in contrast to the guideline from developing countries, which utilizes uterine massage as a preventive measure for PPH [81,86].

All guidelines highlight the significance of active management of the third stage of labor for the prevention of PPH. This requires the administration of oxytocin as the standard measure for successful third placental delivery [1,18,60,80,82,84,87,88,89].

#### 3.5.1. Prevention

The management of PPH includes appropriate prevention of possible events and treatment of PPH incidents. Timely and suitable prophylactic measures are helpful in preventing PPH. Moreover, awareness of possible bleeding after delivery keeps healthcare professionals ready for PPH [16,60].

Prevention of PPH starts with careful assessment of risk factors for all pregnant women antenatally and/or intrapartum [21]. Labor/delivery management plans must be developed/modified based on particular women’s risks. Patients with known risk factors for PPH should give birth in a setting with available and appropriate pharmacotherapy and a blood bank [21,60]. As discussed above, clinicians should be aware of the proper estimation of blood loss and keep in mind that visual approximation is not accurate [21,64]. Relevant healthcare professionals (anesthesiologists, obstetricians, midwives) with appropriate experience/expertise should be alerted of PPH [21].

General recommendations for PPH prevention include active management of the third stage of labor via the utilization of uterotonics and controlled cord traction [21,60,80,81]. However, if a skilled healthcare professional is not available for delivery, controlled cord traction is not recommended [80]. Oxytocin (10 IU) is the pharmacotherapy of choice and is recommended intramuscularly or intravenously for the prevention of PPH in both vaginal delivery and cesarean section [60,80]. Utilized as a part of active management of the third stage of labor, it has proven effective in the prevention of PPH.

In cases when oxytocin is not available or “its quality cannot be guaranteed”, other uterotonics are recommended: injectable ergometrine/methylergometrine 200 μg, oral or rectal 400–600 µg misoprostol, or carbetocin 100 µg IM/IV is recommended for the prevention of PPH [60,80]. In settings where experienced midwives are not available to administer injectable uterotonics (for example, in underdeveloped countries), oral misoprostol could be helpful for the prevention of PPH [60,80].

Some guidelines from developing countries also recommend uterine massage for the prevention of PPH [81,86], while algorithms from developed countries do not recommend it [21,80]. Nevertheless, postpartum assessment of uterine tonus via abdominal palpation “is recommended for all women” [80].

#### 3.5.2. Management

Early diagnosis is vital in the management of PPH. Confirmation of PPH diagnosis is challenging as it is always based on blood loss quantification cut-offs of 500 mL for vaginal delivery and 1000 mL for cesarean delivery [90]. Immediately after confirmation of PPH diagnosis, specific management/therapy should be initiated based on a national guideline or an institutional algorithm, and a multidisciplinary team should be activated (Table 3) [1,51]. Simultaneous team-based actions to monitor vital signs, laboratory work-up, and therapy must be taken.

Although uterine atony is the most common cause of PPH, careful examination must be performed to define the exact cause of PPH [1,6]. It will ensure a timely and appropriate therapeutic approach [1]. As “tissue” and “trauma” are the second most common causes of PPH, specific manipulations must be performed to rule out retained placental tissue in the uterine cavity, hematomas, uterine inversion, and perineal lacerations under anesthesia when necessary [1,6].

##### Medical Management

The generally recommended first-line uterotonic for the treatment of PPH is intravenous oxytocin [1,6,11,21,43,80,81,91]. Oxytocin is a medication administered to reinforce the action of the naturally produced oxytocin (Table 4). Similar to natural oxytocin, this medication has a rapid effect and causes uterine contraction [92]. Oxytocin acts immediately after intravenous injection administration and within 5 min after intramuscular injection [93]. However, the medication may result in several side effects: hemodynamic instability (hypotension, tachycardia, and myocardial ischemia), nausea, vomiting, headache, and flushing [94]. The advantage is that oxytocin is inexpensive and available in most clinical settings, even in low-resource countries.

However, in cases when oxytocin is unavailable or if the hemorrhage does not respond to oxytocin administration, methylergonovine (ergometrine) could be used [1,94]. It is an ergot alkaloid, which binds adrenergic receptors in the myometrium and causes a strong uterine contraction [1]. Methylergonovine is recommended as a second-line uterotonic [15,94]. It is available for intramuscular and intravenous administration (Table 4). It can be considered a beneficial option due to its rapid bioavailability and long half-life [1,94,95]. The most common side effect of methylergonovine is vasoconstriction, followed by hypertension and headaches [94].

Another option to control atonic PPH is carboprost, a synthetic prostaglandin analog of prostaglandin F2α [1,93,94]. Unlike natural prostaglandins with rapid metabolism, carboprost is a longer-acting pharmaceutics with a half-life of 2 h [93,94,96]. Moreover, carboprost is relatively expensive compared to oxytocin and ergometrine, thus not always available in LMICs.

Misoprostol is a synthetic prostaglandin E1, which is a potent uterotonic [1,93,96]. It is available in tablets and is commonly used for the induction of labor, and can be administered orally, vaginally, or rectally [1]. Misoprostol acts relatively fast, within 9–15 min after oral administration. Vaginal/rectal administrations of misoprostol result in slower action onset [94]. Misoprostol is widely recommended for PPH management [80]. However, in cases of PPH, to avoid the medication flashing out due to bleeding, it is usually administered rectally. The medication does not require additional supplies for administration, is relatively cheap, and, therefore, is considered excellent for PPH management in low-resource settings [1]. Compared to oxytocin, misoprostol is connected to more side effects such as fever, chills, nausea, and vomiting [1,94].

An alternative opportunity to improve hemostasis in PPH is the administration of tranexamic acid [65,80,97]. Tranexamic acid inhibits the enzymatic action of plasmin on fibrin and, thus, has an anti-fibrinolytic effect and stabilizes a blood clot. As soon as PPH is diagnosed, guidelines suggest “early use” of intravenous tranexamic acid (100 mg/mL) within 3 h after delivery [65,80,81]. The other resources recommend that women with PPH “receive 1 g tranexamic acid intravenously as soon as possible” after delivery [97,98]. If bleeding continues, the second dose should be administered “after 30 min or restarts within 24 h since the first dose” [5,97]. Tranexamic acid helps in reducing maternal deaths due to maternal bleeding and could improve outcomes of PPH among women in low-resource settings [5,80].

##### Nonsurgical Management

Uterine massage is considered a non-medical and non-surgical step in the conservative management of PPH. Unlike in the case of algorithms for PPH prevention, all guidelines recommend uterine massage for the therapy of PPH [11,21,80,81,91].

Some resources suggest a urinary Foley catheter placement to “monitor urine output for response to resuscitation and decompress the bladder” [1]. Since Foley catheter placement is inexpensive, this step might be beneficial for LMICs and useful in the complex of events for PPH management.

If pharmacological therapy fails, the RCOG guideline highlights that “surgical interventions should be initiated sooner rather than later” when pharmacological management is not effective in controlling PPH [21]. As an appropriate “first-line ‘surgical’ intervention for most women”, intrauterine balloon tamponade is recommended in cases when uterine atony is the main cause of PPH [21,80]. Many studies have shown the effectiveness of intrauterine balloon tamponade, which allows a significant reduction in invasive treatment rates [99]. Different types of balloons are available, including novel prototypes that can be used for patients who do not respond to uterotonic medications [100,101,102,103]. The Bakri balloon is one of the well-known types used to control PPH, which, in comparison with other conservative interventions, demonstrated significant advantages in clinical application [104,105]. The Bakri balloon is a good option for PPH management in a low-resource setting as it requires minimal resources and basic training and possesses high effectiveness in fertility preservation [102,105]. However, the disadvantage of the method is also reported by research suggesting that massive bleeding and severe PPH could be associated with intrauterine balloon failure [99].

In addition, the use of chitosan-covered gauzes was reported by several studies [106,107,108]. Utilization of chitosan-covered gauzes in the management of PPH has shown comparable results to balloon tamponade [102,106,107]. Studies highlight the lower price of the gauzes compared to the Bakri balloon, which makes them a potentially cost-effective alternative [102,106,107].

Another new tool utilized to facilitate the management of PPH is a non-pneumatic anti-shock garment (NASG) [109]. It is a first-aid device in cases of hypovolemic shock and could be mobilized to save women’s lives in PPH. The FIGO guideline recommends the use of the NASG as a temporary measure until appropriate care for PPH and hemorrhagic shock is available [80,104,109]. However, this tool’s availability in a resource-limited setting might be questionable.

If available in specialized hospitals, uterine artery embolization (UAE) can be used as a management measure for PPH [19,80]. Although it requires a specific facility, equipment, and trained physicians, it is one of the effective measures in the management of bleeding. Utilization of the UAE procedure for PPH management results in significantly lower blood loss, and the rates of bleeding control range between 75 and 100% [1,3,110].

##### Surgical Management

In some cases, the relatively noninvasive treatment options do not help to achieve hemostasis [111]. The next advanced step in PPH management, following unsuccessful pharmacological therapy, includes surgical interventions. Those are the application of compression suture techniques, uterine artery ligation, hysterectomy, and bilateral internal iliac artery ligation (Table 5) [4,21,43,80,81]. Those techniques are well developed; however, they require skilled physicians and necessary consumables, which may not be available in a resource-limited setting.

Uterine compression sutures are very effective in achieving uterine hemostasis, especially in cases of PPH due to uterine atony [111,112,113,114]. Various uterine compression sutures have been invented and introduced into clinical practice in the last three decades (Table 5) [111,113]. B-Lynch is one of the most popular uterine sutures widely used all over the globe [98,99,100]. While none of the specific compression suturing procedures show superiority [80,111,113,115], uterine compression sutures are a good conservative option to control PPH with a fertility-sparing opportunity [113,116]. The disadvantage is the need to perform abdominal incisions regardless of whether the delivery was vaginal or through cesarean section [113]. Any of the available suturing techniques could be used in cases of an emergency need to stop PPH, based on the physicians’ experience and resource availability.

**Table 5:** Options for surgical management and indications.

Surgical Intervention	Indication	Notes	References
Uterine compression sutures: B-Lynch, Pereira, Hayman, Cho, Ouahba, Hackethal, Meydanli, etc.	Ineffective pharmacological methods of atonic uterine bleeding without signs of DIC syndrome and Couvelaire uterus	A sequence of interventions depends on the clinical case and a surgeon’s experience; Neither technique has advantages; FIGO recommends uterine artery ligation as one of the fastest methods for controlling PPH; Internal iliac artery ligation might help to avoid hysterectomy in cases of PPH due to uterine atony	[50,80,111,113,114,115]
Urerine artery ligation: Waters’, O’Leary, Tsirulnikov’s sutures	Ineffective uterine compression sutures		
Internal iliac artery ligation	One of the options for management is cases of ineffective uterine compression sutures		
Subtotal hysterectomy	Atonic uterine bleeding without signs of DIC syndrome, uterine cervical lacerations. In cases of ineffective uterine compression sutures, uncorrectable uterine inversion, and placenta increta/percreta		
Total hysterectomy	Atonic uterine bleeding with signs of DIC syndrome, uterine rupture, Couvelaire uterus, and cases of low-lying placenta and placenta previa increta/percreta		
Internal iliac arteries ligation + total hysterectomy	Atonic uterine bleeding with signs of DIC syndrome, uterine rupture, coagulopathy	In cases of traumatic PPH, bilateral internal iliac artery ligation reduces risks of surgical site bleeding and facilitates total hysterectomy	

DIC—disseminated intravascular coagulation; FIGO—The International Federation of Gynecology and Obstetrics; PPH—postpartum bleeding.

The uterine artery ligation procedure is based on blockage of the blood supply to the uterus and, thus, has a hemostatic effect [1,50,117]. It helps to effectively manage PPH and, in many cases, is used simultaneously with uterine compression sutures [116,117]. Uterine artery ligation was first described by Waters in the 1950s and O’Leary in the 1960s [1,50]. It assumes ligation of the ascending branch of the uterine artery [1,50]. After this procedure, the blood flow to the uterus will remain stable due to the blood supply through the ovarian artery and other collaterals [50]. The advantages and disadvantages of uterine artery ligation procedures are similar to those following the uterine compression suture application. In rare cases, it may lead to uterine necrosis [118].

The next step utilized in cases of ineffective uterine compression sutures and uterine artery ligation is a hysterectomy, which is considered one of the effective management options for PPH. It is useful for all types of PPH that have failed other approaches. If performed timely, without unnecessary delay, a hysterectomy can potentially save patients’ lives [80,116]. However, emergency hysterectomy must be discussed critically and performed by skilled professionals [116]. The disadvantage of this intervention is the loss of fertility, and has a serious impact on the physical and mental health of women. Moreover, postpartum hysterectomy is considered a “near miss” event and is associated with high rates of severe maternal morbidity like additional hemorrhage, trauma, and increased mortality [4,44,116].

In complex cases, hysterectomy is used in PPH management together with bilateral internal iliac artery ligation. Bilateral ligation of the internal iliac arteries for the management of PPH was first described at the end of the 19th century [50]. For many decades, it was considered the main vascular technique for PPH management. It assists in the management of PPH caused by coagulopathy. However, this technique necessitates special expertise that is not possessed by all obstetricians. Moreover, internal iliac artery ligation requires extensive retroperitoneal dissection [1]. Like the other vessel ligation techniques in PPH therapy, it is performed through a transabdominal approach [50]. Similar to uterine artery ligation, the internal iliac artery ligation procedure must be performed bilaterally due to the existence of numerous collateral blood supplies between the internal iliac artery and other uterine vessels [50,116]. A recent study suggests that internal iliac artery ligation leads to a significant decrease in the ovarian reserve [119].

Overall, a standardized and adequate management of PPH, including an objective assessment of blood loss volume and the use of a “treatment bundle,” is recommended for all women [60].

##### Resuscitation

The general resuscitation care for women with PPH is similar to the approach for patients with bleeding and hemorrhagic shock [75]. Resuscitation strategies for hemorrhagic shock indicate bleeding control and blood volume replacement [21,65,75].

Primary actions should ensure coordination of patient care by a multidisciplinary team involving senior members and including simultaneous action of the team for identification of cases of PPH and its timely cure, emergency laboratory blood testing (blood cell counts, coagulation tests, blood typing, and cross-testing, etc.), availability of blood derivatives, provision of blood volume replacement, central line access, and availability of oxygen therapy [21,65,75].

Transfusions are an essential part of PPH treatment, especially in the most severe cases, especially in PPH caused by preexisting coagulation disorders (“thrombin”) [6,120]. Blood volume replacement must be performed considering the fact that blood loss is “often underestimated” [21]. The appropriate transfusion in case of massive PPH should include the fast administration of crystalloids to ensure adequate circulating blood volume (Table 6) [80,120,121].

For intravenous infusion, isotonic crystalloids are preferred as a part of emergency care in PPH [1,11,80,91,119,120,122]. Furthermore, the administration of coagulation factors is useful for the maintenance and reinforcement of blood clotting in PPH [21,65,98]. The transfusion of red blood cells guarantees proper tissue oxygenation, while fresh–frozen plasma platelets ensure the management of coagulopathy [21,65,114]. Fibrinogen concentrate or cryoprecipitate may be very helpful in restoring the coagulation process; however, it depends on availability [90]. Compared to cryoprecipitate and fresh–frozen plasma, which require special storage and thawing before use, fibrinogen concentrate infusion has advantages that include convenient storage, easy administration, standardized fibrinogen concentration, and low risk of complications [5,65,98].

Administration of appropriate and timely resuscitation directly depends on the availability of fluids, laboratory test guidance, blood components, and trained personnel. In low-resource settings, due to specific requirements for preparation and storage, these blood components (fresh–frozen plasma, erythrocyte and platelet masses, cryoprecipitate) could be unavailable.

### 3.6. Challenges in the Management of Postpartum Hemorrhage in Developing Countries

PPH remains the number one cause of maternal mortality in developing countries. Despite advances in the prevention and management of PPH, currently, access to well-equipped and fully-staffed facilities is low in LMICs [123]. Developing countries’ healthcare systems face multiple challenges that may impact PPH management at policy, facility, and community levels [17,89]. These challenges include deficits in the budget, appropriate facilities, equipment, and consumables; lack of trained healthcare professionals; absence of information from current evidence and discrepancies in guidelines; and problems with diagnostic and management strategies [37,80].

The mentioned barriers could be addressed by providing an empowering environment through supportive policies, access to PPH care, planning supplies, allying strategies, and utilization of guidelines and protocols for PPH management [17,89]. Challenges related to the lack of evidence-based information at the local level could be addressed by the implementation of adaptation of international guidelines and the WHO recommendations on PPH management [11,60,80,91].

In order to decrease the rate of PPH and subsequently reduce maternal mortality caused by PPH in low-resource settings, it is essential to improve healthcare professionals’ education and knowledge via the implementation of proper practices on the utilization of uterotonics and effective PPH prevention techniques [124].

Despite the availability of clear recommendations regarding PPH diagnosis and management, the uptake of the guidelines was poor [125]. There are several barriers to implementation, including limited availability of staff, deficiency of relevant knowledge and skills, “lack of engagement from health care providers, and professional attitudes that discouraged task sharing” [125,126]. To address these challenges, researchers designed “a cluster-randomized trial to assess a multicomponent strategy for the detection and treatment of postpartum hemorrhage after vaginal delivery” [125]. In 2023, the results of the E-MOTIVE trial were released. E-MOTIVE was an international study that was conducted across 80 secondary-level hospitals in Kenya, Nigeria, South Africa, and Tanzania [125]. The aim of the trial was to assess a multicomponent clinical intervention for women with PPH after vaginal delivery. The intervention included the use of a “calibrated blood-collection drape” for early detection of PPH and a bundle of first-response treatments [125,127]. This bundle of treatment includes uterine massage (M), oxytocic drugs (O), tranexamic acid (T), intravenous fluids (IV), examination, and escalation (E) for early detection of PPH—E-MOTIVE [125,127].

The E-MOTIVE intervention showed that early detection of PPH and use of “bundled treatment” resulted in a 60% reduction of risks for severe PPH, laparotomy for bleeding, or death from bleeding, compared to conventional care among women after vaginal delivery in Kenya, Nigeria, South Africa, and Tanzania [74,125,127]. This benefit of the method was attributed to improvements in the detection of PPH and the use of the WHO first-response bundle in the hospitals in the intervention group [60,125,127].

Raising awareness and training local obstetric teams are confirmed as better options than relying on sophisticated technological resources that are almost never present in low-resource circumstances. Simulation training on PPH management is encouraged and proven to be effective in the education and coaching of healthcare professionals involved in the labor/delivery facilitation process [80]. This is of special importance for LMICs, where restrictions on additional resources might be replenished by trained providers. Moreover, in addition to national guidelines, each hospital should have a PPH management algorithm posted in the labor ward for healthcare providers’ convenience [80].

There are some justified technologies for the prevention and treatment of PPH, which do not require extensive budgeting and could be used in rural areas by staff with limited skills after training [123]. These PPH prevention and treatment measures include oxytocin injection after fetal delivery, a common application of active management for the third stage of labor facilitation, and the administration of oral or rectal misoprostol; and the hydrostatic balloon catheter to control PPH [80,123]. Experience of the past decades confirmed that active management applied during placental delivery (the third stage of labor) is one of the most efficient approaches to prevent PPH [123]. This method does not require additional resources; only appropriate training must be conducted among involved healthcare providers (physicians, nurses, midwives). Up-to-date techniques and approaches developed to combat PPH should be available in low-resource countries with high rates of PPH to enable a decrease in maternal mortality.

Moreover, in LMICs, to minimize unfavorable outcomes, an accurate pregnant patient selection should be made, and women from level I and rural clinics who are at high risk of PPH must be transferred to higher-level centers (level II-IV, depending on the particular case) in a timely manner. For patients who develop PPH, a timely transfer from the labor suit to the emergency care unit must be considered at the particular hospital level [128]. This will ensure proper resuscitation and management.

Appropriate management of PPH is a working instrument enabling the reduction of maternal mortality and could have a positive impact on health equity and improving outcomes among women in LMICs [80]. In many developing countries, maternal morbidity and mortality associated with PPH were improved after the implementation of standardized protocols and timely and purposeful interventions [43,83,87,89].

## 4. Conclusions

Despite the progress in medical care and the application of evidence-based approaches and methods, PPH remains a huge problem in obstetrics emergencies worldwide, demanding vigilant monitoring and prompt intervention. The high burden of PPH and subsequent maternal mortality is especially alarming in the developing world. Our review underscores the diverse etiologies and risk factors associated with this condition, emphasizing the need for a comprehensive approach to both prevention and management. A unified definition is important for the objective estimation of PPH and the timely initiation of proper care. Effective strategies, timely treatment, and the use of evidence-based protocols are essential in reducing the incidence of severe outcomes and improving patient safety. Evidence-based international guidelines should serve as an integral part of appropriate management. LMICs have no opportunities to use modern and complex equipment/medications but rely on their physicians’ training and experience. The proposed international algorithms and guidelines are suggested for use in the developing world; however, some amendments are required based on the availability of resources/expertise. Enhancing our understanding of specific local circumstances, international support in specialists’ training, and the provision of evidence-based approaches by the WHO, FIGO, and other expert boards may assist in improving the management of PPH in LMICs and contribute to safer childbirth.

## Figures and Tables

**Table 2:** Classification of postpartum hemorrhage by stage [5,10,21,26].

Stage	Blood Loss (mL)
Stage 0	Vaginal delivery: <500 Cesarean delivery: <1000
Stage 1 (mild)	Vaginal delivery: 500–1000 Cesarean delivery: 1000
Stage 2 (moderate)	1000–1500
Stage 3 (severe)	>1500

**Table 3:** Actions based on the etiology of postpartum hemorrhage.

“Tonus”	“Trauma”	“Tissue”	“Thrombin”
Uterine massage	Per speculum examination	Uterine cavity examination	Transfusion of erythrocyte mass, FFP, platelets
2. Uterotonics	2. Suturing of lacerations	2. Retained placental or amniotic tissue removal	2. Transfusion of recombinant coagulation factors
3. Bimanual uterine compression	3. In case of uterine inversion—reparation of the uterus under anesthesia		
4. Balloon tamponade	4. In case of uterine rupture—laparotomy and surgical management based on the situation		
5. Surgical hemostasis			

Abbreviation: FFP—fresh frozen plasma.

**Table 4:** Uterotonic medications [21,26,60,65].

Medication	Mechanism of Action	Dose	Route of Administration
Oxytocin	Direct stimulation of oxytocin receptors	- 10 IU for routine PPH prevention	- IM
		- 20–40 IU for PPH treatment	- IV rapid (4–5 min), then 7–15 IU per hour once uterine tonus is achieved
Carbetocin	Stimulation of oxytocin receptors	100 μg	IM or slow IV injection (≥30 s)
Ergometrine/methylergonovine	Activation of adrenergic and dopaminergic receptors of uterine smooth muscles and vascular smooth muscle layers	250 μg	IM or IV over 1 min (rare circumstances)
Misoprostol	Stimulation of prostaglandin receptors	200–400–600 μg	Sublingual, oral, rectal
Carboprost	Stimulation of oxytocin receptors	250 μg	IM

Footnotes: IM—intramuscular; IV—intravenous; IU—international units.

**Table 6:** Resuscitation therapy and blood product transfusion [21,26,65,121].

Transfusion Substance	Dosage	Indication
Crystalloid fluids	Infusion of up to 2000 mL (1–2 mL crystalloid for each 1 mL blood loss)	Active bleeding
Colloid fluids	Infusion of up to 1500 ml	Active bleeding
Fresh-frozen plasma	15–20 mL kg^−1^, dosing and administration under the guidance and control of coagulation tests	APTT and PT/INR 1.5 times prolonged compared to normal
Erythrocytes mass	Group-specific, number of units depends on the hemoglobin level and red blood cell count	Hemoglobin concentration is <70 g/L or 7 g/dL^−1^
Thrombocytes mass	One pool or 5–10 mL kg^−1^	Drop of thrombocytes count to less than 50–75 × 10^9^ and in case of continuous bleeding
Fibrinogen concentrate	25–50 mg kg^−1^(1 g in 50 mL injection water)	Fibrinogen concentration ≤ 2 g/L
Cryoprecipitate	Two pools or 1 unit per 5–10 kg body weight or 4–6 mL kg^−1^	Fibrinogen concentration is less than 2 g/L

Footnote: APTT—activated partial thromboplastin time; PT—prothrombin time; INR—international normalized ratio.
